# Supplementary material for: Complexome profiling of the Chlamydomonas psb28 mutant reveals TEF5 as an early PSII assembly factor
Source: Plant Cell. 2025 Mar 17;37(6):koaf055. doi: 10.1093/plcell/koaf055 (PMC12164590; doi:10.1093/plcell/koaf055)
Supplement: koaf055_Supplementary_Data [file koaf055_supplementary_data.zip › psb28_interactive.zip!/plots/intensity/qualityPlots/peptides/GGH2_Cre06.g275050.t1.1.html]

### Cre06.g275050.t1.1

Trivial name: GGH2  
SeqCoverage [median]: 9.00  
Mass: 41.40 kDa  
  
 

| Sequence | MissedCleavage | Mass [Da] | wtIntensity | muIntensity | Score |
| --- | --- | --- | --- | --- | --- |
| MSQEVANFFVSEAR | 0 | 1613.75 | 0.00 | 1028.11 | 111.63 |
| SGAAYISTLEGR | 0 | 1223.61 | 560.73 | 0.00 | 78.65 |
| VLSLSLDK | 0 | 873.52 | 0.00 | 4091.54 | 73.62 |
|  |  |  | 560.7 | 5119.6 |  |
